# The National Diabetes Education Program Evaluation Framework: How to Design an Evaluation of a Multifaceted Public Health Education Program

**Published:** 2008-09-15

**Authors:** Joanne Gallivan, Rachel Greenberg, Clarice Brown

**Affiliations:** National Diabetes Education Program, National Institutes of Health; Washington, District of Columbia; Surveys and Epidemiology Services Division, Social and Scientific Systems, Silver Spring, Maryland

## Abstract

The National Diabetes Education Program, cosponsored by the National Institutes of Health and the Centers for Disease Control and Prevention, employs mass media communications, public-private partnerships, and dissemination of information and education tools to address the diabetes epidemic in the United States. The program's goal is to help reduce the morbidity and mortality from diabetes and its complications by improving the treatment and outcomes for people with diabetes, promoting early diagnosis, and preventing onset of diabetes. Evaluation is an integral component of the National Diabetes Education Program's planning and implementation process. The program's evaluation is based on the Centers for Disease Control and Prevention's Framework for Program Evaluation in Public Health, which has guided program planners and evaluators in developing measurable short-term, midterm, and long-term outcomes. We describe how the National Diabetes Education Program has applied the evaluation framework, demonstrating how multifaceted health communications programs can design program evaluations to answer key questions about program processes and outcomes.

## Introduction

Diabetes was the seventh leading cause of death in the United States in 2006; approximately 233,619 deaths were attributed to the disease (1). In 2007, an estimated 23.6 million people had diabetes, representing 7.8% of the US population (1). Of these, 17.9 million cases were diagnosed, leaving 5.7 million people unaware they had diabetes. Another estimated 57 million adults aged 20 years or older had prediabetes in 2007, putting them at increased risk for diabetes (1).

The US Department of Health and Human Services launched the National Diabetes Education Program (NDEP) in 1997 to improve diabetes management and to help reduce the morbidity and mortality from diabetes and its complications. The leading federal government program that promotes diabetes prevention and control, NDEP is cosponsored by the National Institute of Diabetes and Digestive and Kidney Diseases of the National Institutes of Health (NIH) and the Division of Diabetes Translation of the Centers for Disease Control and Prevention (CDC). The aim of NDEP is to improve the treatment of diabetes and its complications, to promote early diagnosis, and to prevent the onset of diabetes (2). To reach these goals, NDEP has formulated the following program objectives:

- Increase awareness of the seriousness of diabetes, its risk factors, and strategies for preventing diabetes and its complications among groups at risk.
- Improve understanding about diabetes and its control and promote better self-management behaviors among people with diabetes and their social supporters.
- Improve health care providers' understanding of diabetes and its control and promote an integrated approach to care.
- Promote health care policies that improve the quality of and access to diabetes care.
- Reduce health disparities in racial and ethnic populations disproportionately affected by diabetes.

### Guiding principles of NDEP implementation and evaluation

Four central principles guide NDEP's planning, implementation, and evaluation activities. These principles are based on effective approaches used by the National High Blood Pressure Education Program, the National Cholesterol Education Program, and other education programs during the past 30 years (3).

The first principle is that the program must be based on scientific evidence, including epidemiologic, clinical, and demonstration studies. This evidence shows that much of the illness and death associated with diabetes and its complications can be prevented or delayed by aggressive treatment with diet, physical activity, and pharmacologic approaches that help to normalize blood glucose levels, blood pressure, and lipids (4). Research shows that type 2 diabetes can be prevented or delayed through modest weight loss and regular physical activity (5). NDEP translates the science of diabetes prevention and treatment into public, patient, and professional education messages and materials, community intervention tool kits, and awareness campaigns. These are disseminated through NDEP's publications clearinghouse, its Web sites, and its partners.

The second principle is that an effective education program must involve various organizations that operate in partnership to achieve program goals and objectives (3). A component of NDEP is its partnership network of approximately 200 public- and private-sector organizations. Program partners disseminate and promote NDEP's mass media campaigns and educational messages through national, state, and local communication channels. They also provide guidance on developing appropriate messages and strategies by participating in audience-specific work groups. These work groups meet monthly via telephone conference calls and every 2 years at face-to-face meetings, where they develop and review the progress of their respective strategic plans, media messages, educational products, and community channel activities.

The third principle is that public, patient, and professional education must use effective communication strategies to reach selected target audiences (3). NDEP offers a wide range of resources to support 2 major public education campaigns: "Control Your Diabetes. For Life." and "Small Steps. Big Rewards. Prevent Type 2 Diabetes." Each campaign offers partners a wealth of tools — brochures, tip sheets, public service advertising, health care provider tool kits, community intervention guides, and more — for conducting outreach activities in communities across the country. Consumer materials are carefully tailored for groups at highest risk for diabetes, including older adults, African Americans, American Indians, Alaska Natives, Hispanics and Latinos, Asian Americans and Pacific Islanders, and women with a history of gestational diabetes. Many NDEP educational and promotional materials are available in as many as 15 languages, including Spanish and Tagalog.

The fourth principle is that evaluation must be an integral component of program planning and implementation and must be used as part of an iterative process of re-planning and refining program activities (2). This principle has inspired a comprehensive approach to NDEP evaluation, encompassing both process and outcome evaluation. The process evaluation monitors program implementation and short-term effects. NDEP uses the resulting findings to identify areas in need of midcourse correction or continuation. The outcome evaluation focuses on the midterm and longer-term effects of NDEP's efforts, particularly NDEP's and partner organizations' promotion and outreach activities to target audiences. Progress on these midterm and longer-term outcomes is measured by tracking changes in consumer and health care provider awareness, knowledge, attitudes, beliefs, and behaviors about diabetes prevention and control.

### Framework for Program Evaluation in Public Health

NDEP bases its program evaluation on the Framework for Program Evaluation in Public Health (6). The framework, developed to help ensure that public health programs "remain accountable and committed to achieving measurable outcomes" (6, p. 1), encompasses 6 steps:

- Engage important stakeholders
- Describe the program
- Focus the evaluation
- Gather credible evidence
- Analyze and interpret data
- Use the findings

We describe how NDEP has applied the evaluation framework, providing a case study of how a multifaceted public health communications program can design program evaluations to help answer key questions on program processes and effects.

## Step 1: Engage Important Stakeholders

Engaging stakeholders is central to establishing a common frame of reference about the program and the key evaluation questions to be asked, and to ensuring that the evaluation findings are used for program improvement. In designing its evaluation, NDEP involved members of its partnership network, which includes the leading national organizations of health professionals and volunteers concerned about diabetes, state diabetes prevention and control programs, community health professionals and organizations concerned about diabetes, and representatives from private industry. Members of the Partnership Network participate in NDEP's Steering and Operations committees and in the program's 10 work groups. NDEP engaged key stakeholders in several ways.

### Stakeholders involved in program operations

These stakeholders are engaged in program evaluation through the program's Operations Committee. The committee meets twice per year to review the findings of process evaluation activities, such as NDEP's semiannual Partner Activities Survey, and to review progress made toward achieving the program's strategic planning goals.

### Stakeholders served or affected by the program

These stakeholders are the approximately 200 members of the NDEP Partnership Network that includes all the partner organizations represented on the program's Steering Committee, members of the work groups, and the 59 state and territorial diabetes prevention and control programs. These partners help promote and disseminate NDEP's awareness campaigns, educational materials, and resources on diabetes prevention and control.

NDEP engages these stakeholders in program evaluation through the program's semiannual Partner Activities Survey, which asks partners to report on their promotion and dissemination efforts related to NDEP campaigns. Partners are sometimes engaged in NDEP's formative research activities, such as pretesting or pilot-testing NDEP products before they are produced in final form.

### Primary users of the evaluation

These stakeholders include NDEP's Executive Committee, composed of the directors of the diabetes divisions at NIH and CDC, the chairs of the steering and operations committees, and the program directors of NDEP at NIH and CDC. NDEP formed an Evaluation Work Group that provides guidance on program evaluation design and implementation. The roles and responsibilities of the Evaluation Work Group members include

- Reviewing and updating the evaluation plan.
- Providing impact and outcome data from the organizations they represent for NDEP evaluation efforts.
- Identifying strategies to fill gaps in data for the evaluation plan.
- Reviewing and providing counsel for NDEP surveys and other research tools.

## Step 2: Describe the Program

In the CDC framework, a consensus program description helps develop the evaluation focus, associated indicators, and data sources. A strong, comprehensive program description also integrates the planning and evaluation process, as was the case for NDEP.

NDEP's program description emerged from an extensive review of the literature on health behavior research on diabetes and from strategic planning meetings with experts in diabetes education and representatives of stakeholder groups. The review of the literature and the strategic planning meetings provided direction for developing the program's first strategic plan (NDEP, unpublished data, 1997). The plan formulated a statement of need for the program and presented key objectives, target audiences, messages, strategies, and tactics it would employ.

**Figure 1.**
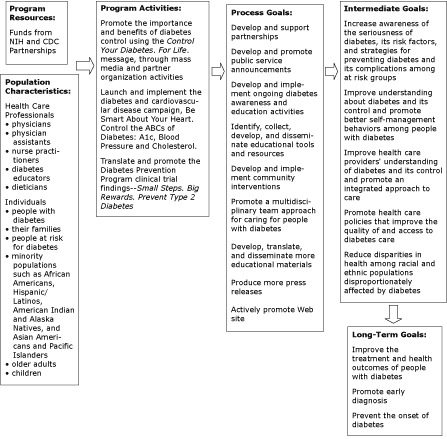
National Diabetes Education Program Conceptual Framework. Abbreviations: NIH, National Institutes of Health; CDC, Centers for Disease Control and Prevention.

The NDEP Evaluation Work Group provided guidance on developing outcome measures for evaluating the program by prioritizing a 3-tiered list of outcome measures for each target audience. From the list of prioritized outcome measures, the work group developed a conceptual program evaluation framework that included an overview of program resources, program activities, process goals, and intermediate and long-term goals (Figure 1).

**Table:** 

The conceptual framework begins with Program Resources and Population Characteristics: Program Resources are funds from the National Institutes of Health and the Centers for Disease Control and Prevention, as well as Partnerships. The Population Characteristics are in two categories: Health Care Professionals (physicians, physician assistants, nurse practitioners, diabetes educators, and dietitians) and Individuals (people with diabetes; their families; people at risk for diabetes; minority populations such as African Americans, Hispanic/Latinos, American Indian and Alaska Natives, and Asian Americans and Pacific Islanders; older adults; and children).
The Program Resources and Population Characteristics influence Program Activities, which include the following activities:
Promote the importance and benefits of diabetes control using the *Control Your Diabetes. For Life.* message, through mass media and partner organization activities Launch and implement the diabetes and cardiovascular disease campaign, *Be Smart About your Heart. Control the ABCs of Diabetes: A1c, Blood Pressure and Cholesterol* Translate and promote the Diabetes Prevention Program clinical trial findings — *Small Steps. Big Rewards. Prevent Type 2 Diabetes*
The Program Activities lead to the Process Goals in these ways:
Develop and support partnerships Develop and promote public service announcements Develop and implement ongoing diabetes awareness and education activities Identify, collect, develop, and disseminate educational tools and resources Develop and implement community interventions Promote a multidisciplinary team approach for caring for people with diabetes Develop, translate, and disseminate more educational materials Produce more press releases Actively promote Web site
The Process Goals lead to the Intermediate Goals, which include the following:
Increase awareness of the seriousness of diabetes, its risk factors, and strategies for preventing diabetes and its complications among at-risk groups Improve understanding about diabetes and its control and promote better self-management behaviors among people with diabetes Improve health care providers' understanding of diabetes and its control and promote an integrated approach to care Promote health care policies that improve the quality of and access to diabetes care Reduce disparities in health among racial and ethnic populations disproportionately affected by diabetes
The Intermediate Goals lead to the Long-Term Goals:
Improve the treatment and health outcomes of people with diabetes Promote early diagnosis Prevent the onset of diabetes

**Figure 2.**
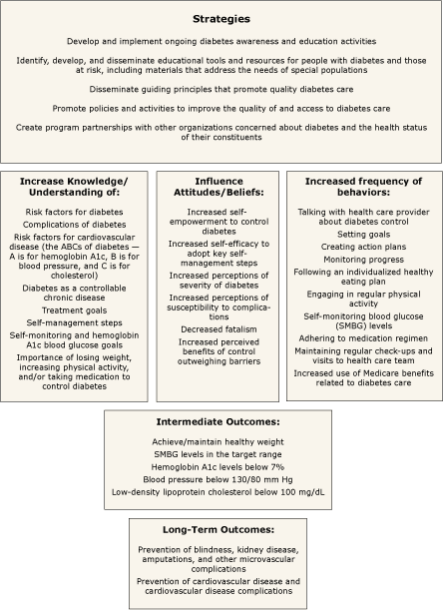
Strategies and Outcomes in Glucose Control

The evaluation work group and staff used the conceptual framework to develop the more explicit logic model. For example, in looking at the program's glucose control component, the conceptual framework enabled NDEP to develop a logic model that included key strategies to increase knowledge, influence attitudes and beliefs, and change behaviors through its mass media messages and educational materials and activities. As shown in Figure 2, the framework allowed NDEP to specify the intended intermediate and long-term outcomes the program was seeking related to blood glucose control among people with diabetes.

**Table:** 

The Program Strategies include the following:
Develop and implement ongoing diabetes awareness and education activities Identify, develop, and disseminate educational tools and resources for people with diabetes and those at risk, including materials that address the needs of special populations Disseminate guiding principles that promote quality diabetes care Promote policies and activities to improve the quality of and access to diabetes care Create program partnerships with other organizations concerned about diabetes and the health status of their constituents
There are 3 types of outcomes for these Program Strategies. The first type of outcome is to Increase Knowledge and Understanding of these key points about diabetes:
Risk factors for diabetes Complications of diabetes Risk factors for cardiovascular disease (the ABCs of diabetes — A is for hemoglobin A1c, B is for blood pressure, and C is for cholesterol) Diabetes as a controllable chronic disease Treatment goals Self-management steps Self-monitoring and hemoglobin A1c blood glucose goals Importance of losing weight, increasing physical activity, and/or taking medication to control diabetes
The second type of outcome is to Influence Attitudes/Beliefs, which results in the following:
Increased self-empowerment to control diabetes Increased self-efficacy to adopt key self-management steps Increased perceptions of severity of diabetes Increased perceptions of susceptibility to complications Decreased fatalism Increased perceived benefits of control outweighing barriers
The third type of outcome is Increased Frequency of the following Behaviors:
Talking with health care provider about diabetes control Setting goals Creating action plans Monitoring progress Following an individualized healthy eating plan Engaging in regular physical activity Self-monitoring blood glucose (SMBG) levels Adhering to medication regimen Maintaining regular check-ups and visits to health care team Increased use of Medicare benefits related to diabetes care
The Intermediate Outcomes are the following:
Achieve/maintain healthy weight ISMBG levels in the target range Hemoglobin A1c levels below 7% Blood pressure below 130/80 mm Hg Low-density lipoprotein cholesterol below 100 mg/dL
The Long-Term Outcomes are the following:
Prevention of blindness, kidney disease, amputations, and other microvascular complications Prevention of cardiovascular disease and cardiovascular disease complications

## Step 3: Focus the Evaluation

As a national education program, NDEP focuses its evaluation efforts on monitoring and assessing what the program's mass media campaigns, educational materials, and other promotional activities can plausibly influence; for example, increasing knowledge of the importance of controlling risk factors for cardiovascular disease in people with diabetes, improving a person's feeling of self-efficacy to take charge of diabetes self-management, or increasing the practice of self-monitoring of blood glucose levels. At the same time, NDEP monitors long-term outcomes, such as prevention of diabetes-related complications, by tracking incidence and prevalence of blindness, kidney failure, amputations, and cardiovascular disease as reported by the National Health Care Surveys conducted by CDC's National Center for Health Statistics (NCHS). The program monitors intermediate outcomes, such as the health status of people with diabetes who have major risk factors that contribute to diabetes complications, using data from the National Health and Nutrition Examination Survey (NHANES) and the National Health Interview Survey (NHIS) conducted by NCHS and CDC's Behavioral Risk Factor Surveillance System (BRFSS). NDEP monitors these trends and uses the results to determine what messages and educational activities the program needs to emphasize.

## Step 4: Gather Credible Evidence

The outcomes specified in the NDEP evaluation lend themselves to survey data collection. Because of budget and regulatory constraints on survey research by federal government programs, NDEP devised a multifaceted approach for measuring program outcomes using survey data. The program uses a combination of existing data available from ongoing federal government and partner organization surveys related to diabetes and supplements these data by conducting its own survey research.

### Monitor and track available data

CDC's NCHS coordinates several surveys that incorporate diabetes-related questions, including NHANES, NHIS, and BRFSS. NDEP has used data from these surveys to track many of its outcome measures, including epidemiologic trends in diabetes and cardiovascular risk factor control as well as public knowledge, attitudes, beliefs, and health behaviors regarding diabetes.

### Add NDEP questions to ongoing health surveys

To fill gaps in national data collection on key NDEP outcome measures, the program successfully negotiated adoption of several questions into the NHANES and NHIS diabetes question sets. Responses to these questions will provide NDEP with data about people with diabetes and people at high risk for diabetes.

### Use relevant data from partners

Since the program's inception, NDEP has worked closely with the American Diabetes Association (ADA), the leading health professional and voluntary organization concerned about diabetes, to coordinate evaluation activities. ADA has a strong market research program that includes annual surveys of people with diabetes. The association also commissions periodic physician surveys and national omnibus surveys to track knowledge, attitudes, and practices related to diabetes among the general public and people with diabetes. ADA routinely consults with NDEP about questions to include in these surveys and shares nonproprietary data with the program. This collaboration has provided NDEP with baseline and benchmark data for tracking changes in knowledge, attitudes, and practices for several short-term outcome measures.

Other partners, such as the American Academy of Nurse Practitioners (AANP), conduct routine surveys of their members about knowledge, attitudes, and practices related to various health conditions. The AANP shared their findings related to diabetes management with NDEP and agreed to assist NDEP with conducting survey research among its members.

### Conduct original survey research

NDEP applied for supplementary funding from the NIH Evaluation Branch, explaining the need for the research and how it would be used in program evaluation. Concurrently, NDEP sought permission from the federal Office of Management and Budget (OMB) to conduct original survey research. Federal government regulations restrict agencies from fielding survey research among 10 or more people without OMB approval. These restrictions are to minimize the "paperwork burden" on the public.

After NDEP received funding from NIH and approval from OMB, a national telephone survey was conducted in 2006. NDEP targeted adults aged ≥45 years, the population at highest risk for diabetes and prediabetes. Because diabetes is more prevalent among minority populations, African American and Hispanic/Latino households were oversampled. The survey results will fill most of the remaining gaps on several key evaluation questions about the public's knowledge of diabetes and prediabetes and will provide direction for shaping educational messages and materials.

### Conduct process evaluation

NDEP supplements the extensive outcome data monitoring detailed above with an ongoing system of process evaluation, including the following:

- Tracking television, radio, and print public service advertising placements (ie, the reach and frequency of messages, in terms of the number of placements [frequency] and the size of the audience [reach]).
- Tracking results of press release dissemination though a clipping service (ie, the number of news stories placed and the geographic location and circulation of the publications).
- Tracking the number of publications ordered from the National Diabetes Information Clearinghouse, NDEP's fulfillment organization, and the number of public inquiries to CDC's Division of Diabetes Translation.
- Gathering monthly Web statistics on the number of unique visits to the NDEP Web site and the number of downloads of the top 100 most popular publications.

Another key element of NDEP's process evaluation involves monitoring and tracking NDEP-related activities conducted by members of the partnership network through a semiannual Partner Activities Survey. This online survey asks partners to report on the types of promotion and dissemination activities they have conducted in the past 6 months for each NDEP priority campaign message. This survey also gathers feedback on partners' satisfaction and challenges with NDEP's campaigns, educational materials, and operations.

## Step 5: Analyze and Interpret Data

NDEP reports the results of its process and outcome evaluations to program stakeholders in many ways. One is through NDEP's annual report on evaluation activities and the latest data on outcome measures, which is presented to the program's steering committee. A summary of this report is incorporated into the steering committee meeting minutes, which are disseminated to the entire partnership network. Similarly, a report on NDEP evaluation is included on the agenda of the partnership network's meetings held every 2 years.

The results of the Partner Activities Survey are compiled and disseminated to all members of the partnership network. For example, recent surveys indicate that most partners who respond to these semiannual surveys conduct activities to promote NDEP's awareness campaigns. Many of them exhibit at conferences and meetings, participate in health fairs, make presentations, or conduct training sessions and workshops to promote NDEP's messages and materials. Nearly all the partners promote NDEP's Web site resources to their colleagues and constituents.

The survey reports also provide data on partner activities to the various work groups, who use the information to assess progress in implementing their strategic plans. NDEP has begun preparing process measure reports on media placements and publications dissemination related to each work group's strategic plan. For example, the report to the Hispanic/Latino work group provides process measures on all of the public service announcements and article placements, the number of Spanish-language publications distributed by the program's clearinghouse, and the number of Web downloads of Spanish-language materials.

In 2001 and 2004, NDEP completed and disseminated progress reports on the program's accomplishments, covering 1997 through 2003. To mark NDEP's 10-year anniversary, the program released a 1997-2007 report in September 2007 (7). This report presented the latest available outcome data on NDEP's priority campaigns and summarized process measures regarding media activities, publications dissemination, and Web site usage. These reports provide members of the partnership network and other NDEP constituents with information about where the program has been and where it is headed.

These 3 examples illustrate that people with diabetes have a better understanding of diabetes and are taking steps to control it:

- People with diabetes have shown a dramatic increase in awareness of one of the key measures of diabetes control, the hemoglobin A1c test. According to surveys by the ADA from 1998 to 2004, awareness of the A1c test doubled from 31% to 60% (7).
- People with diabetes report a significant increase in self-monitoring of blood glucose at least once per day from 39% in 1997 to 63% in 2006. Patients who regularly test for glucose are more engaged and active in the management of their diabetes — a critically important step in taking control (7).
- There are also signs that people with diabetes are taking steps to improve outcomes. Comprehensive control of diabetes — control of A1c, blood pressure, and cholesterol (the ABCs of diabetes) — is a key measure of progress in controlling diabetes. Findings in the 2 most recent NHANES surveys indicate a significant increase in people with diabetes taking medications to control cholesterol and hypertension (7).

To inform the diabetes, health professional, and public health communities about NDEP's activities, accomplishments, and lessons learned, NDEP writes and submits journal articles. NDEP staff members and partners make presentations about the program at health professional and public health meetings. The program has been on the agenda of periodic prevention summits of the US Department of Health and Human Services, where representatives have had the opportunity to address public health professionals about NDEP's activities and progress.

## Step 6: Use the Findings

NDEP uses the evaluation activities and reports to assess progress toward reaching program goals and to make midcourse corrections in program activities. NDEP also updates strategic plans, including those for each work group, every 3 years. Program staff members work closely with each work group to review previous plans, assess the latest outcome and process measures, and then develop updated plans for the next 3-year period.

NDEP updates its data as new data from NHANES, NHIS, and BRFSS become available each year. The program has created a database for tracking results from these surveys and charting trends on key intermediate outcomes. These trend data are reviewed and shared with the evaluation work group, the executive committee, and the operations committee to keep them abreast of changes in outcome measures. NDEP also used the data to help set priorities and allocate program resources for messages and campaigns that need to be reinforced and disseminated to selected target audiences. Similarly, results of the NDEP survey of the public's knowledge, attitudes, and practices related to diabetes are assisting NDEP in understanding its target audiences, specifically people with diabetes, people with prediabetes, and people at risk for diabetes, and to inform the program of key issues to consider in program planning.

NDEP analyzes the results of the semiannual Partner Activities Survey to determine what, if any, changes are needed in program operations to better serve partners' needs. The Partner Activities Survey also has provided insights about the role partners play in promoting and disseminating NDEP messages and what gaps the national program staff needs to fill.

## Conclusion

The CDC Framework for Program Evaluation in Public Health is an effective tool for structuring an evaluation process that has a high probability of producing findings that programs can use for improvement. It has given the NDEP a roadmap for asking key evaluation questions and for identifying practical solutions to obtaining answers. The process also has been helpful in educating NDEP's funders, partners, and other stakeholders regarding useful, feasible, appropriate, and accurate measures for evaluating a multifaceted public and professional education program such as NDEP, which is significantly different from a randomized clinical trial or preintervention/postintervention study, the research model that is more familiar to stakeholders.

Engaging stakeholders in the design and implementation of NDEP's evaluation has been a critical strategy. Without the support, guidance, and contributions of the many individuals and organizations that have been involved in the evaluation process, NDEP would have many more gaps in its evaluation framework.

Developing a logic model of the program and identifying the essential evaluation measures needed to assess program outcomes has helped to streamline the evaluation design and eliminate many irrelevant measures. As the program evolves, NDEP will add new components to the logic model, building on previous elements.

Most importantly, NDEP has taken to heart the iterative nature of evaluation as represented by the CDC framework. The true value of this approach for NDEP will be its attention to continuous refinement of the program's efforts based on evaluation findings — that is, closing the loop between data and action.
